# Circ-SOX4 drives the tumorigenesis and development of lung adenocarcinoma via sponging miR-1270 and modulating PLAGL2 to activate WNT signaling pathway

**DOI:** 10.1186/s12935-019-1065-x

**Published:** 2020-01-03

**Authors:** Nan Gao, Baoguo Ye

**Affiliations:** 1 Department of Thoracic Surgery, China-Japan Union Hospital of Jilin University, Changchun, 130033 Jilin China; 2 Department of Anesthesiology, China-Japan Union Hospital of Jilin University, No. 126 XianTai Road, Changchun, 130033 Jilin China

**Keywords:** Circ-SOX4, miR-1270, PLAGL2, WNT, LUAD

## Abstract

**Background:**

Lung adenocarcinoma (LUAD), a widespread histopathological subtype of lung cancer, is deemed as a malignant tumor with a peak risk of mortality. Emerged as RNA with a loop structure that depleted protein coding ability, circular RNA (circRNA) has been identified as a regulator in cancer progression. Circ-SOX4, identified as a novel circRNA, has not been studied in any cancer yet. Thus, the regulatory function that circ-SOX4 exerts on LUAD development remains obscure.

**Aim of the study:**

This study aimed to investigate the biological function and molecular mechanism of circ-SOX4 in LUAD.

**Methods:**

The expression of circ-SOX4 was detected by qRT-PCR. CCK-8, colony formation, transwell and wound healing assays were performed to explore the biological function of circ-SOX4 in LUAD. The interaction between miR-1270 and circ-SOX41 (or PLAGL2) was confirmed by RNA pull down, luciferase reporter and RIP assays.

**Results:**

Circ-SOX4 was found to be obviously upregulated in LUAD tissues and cells, and knockdown of it inhibited cell proliferation, invasion and migration in LUAD. Furthermore, silenced circ-SOX4 also inhibited LUAD tumor growth. Molecular mechanism assays revealed that circ-SOX4 interacted with miR-1270 in LUAD. Besides, PLAGL2 was confirmed as a downstream gene of miR-1270. Rescue assays validated that miR-1270 suppression or PLAGL2 overexpression countervailed circ-SOX4 depletion-mediated inhibition on cell proliferation, invasion and migration in LUAD. Additionally, it was discovered that circ-SOX4/miR-1270/PLAGL2 axis activated WNT signaling pathway in LUAD.

**Conclusions:**

Circ-SOX4 boosted the development of LUAD and activate WNT signaling pathway through sponging miR-1270 and modulating PLAGL2, which provided a valuable theoretical basis for exploring underlying therapeutic target in LUAD.

## Background

Lung cancer is a common type of malignancy and resulted in the death related to cancer worldwide [1]. The proportion of about 84% lung cancers is non-small cell lung cancer (NSCLC) [2]. However, lung adenocarcinoma (LUAD) is the most common kind of NSCLC with high morbidity and mortality [3]. To develop novel treatments in LUAD, numerous efforts have been made over the past decades. However, the prognosis of LUAD patients remains unsatisfactory. As reported, the five-year-survival rate is under ten percent [4]. In consequence, identification of the effective diagnostic and therapeutic methods is essential for timely diagnosing and treating patients with LUAD [5, 6].

Circular RNA (circRNA) is a particular type of noncoding RNA that contains multiple characteristics, including conservation, stabilization and tissue specific expression in living beings [7–9]. Numerous researches have confirmed the various regulatory mechanisms of circRNAs in cancer progression, like serving as sponges for miRNAs, forming RNA–protein complexes, and modulating the transcription of target genes [10, 11]. Some circRNAs have been illustrated to play a key role in cancer progression. For instance, Hsa-circ_0068871 promotes cell proliferation and migration in bladder cancer by sponging miR-181a-5p [12]. Circ-SETD3 inhibits the growth of hepatocellular carcinoma via acting as a sponge of miRNA-421 [13]. Circ-LDLRAD3 functions as a diagnostic biomarker in pancreatic cancer [14]. There are a series of circRNAs were reported in LUAD. Hsa-circ_0001946 regulates miR-135a-5p/SIRT1 axis in LUAD and enhances cell growth by activating Wnt pathway [15]. Hsa-circ_0006427 functions as a tumor suppressor in LUAD progression [16]. As a novel circRNA, circ-SOX4 has not been studied in LUAD. Therefore, the functional role and underlying mechanism of circ-SOX4 needs to be explored. The function of circ-SOX4 in LUAD was identified through both in vitro and in vivo experiments.

MicroRNAs (miRNAs), consisting 18–25 nucleotides, are a class of small RNAs with no coding ability, and exert essential function in the biological process [17]. As reported, miR-203a-3p facilitates cell proliferation and migration in colorectal cancer by targeting PDE4D [18]. microRNA-744 restrains the aggressive behaviors in glioblastoma by targeting NOB1 [19]. Former studies have illustrated that circRNAs affected tumor development by sponging specific miRNAs [20, 21]. For instance, hsa-circ-0005105 facilitates extracellular matrix degradation of chondrocyte via sponging miR-26a [22]. Hsa-circ-0020397 regulates cell proliferation and metastasis in colorectal cancer by sponging miR-138 expression [23]. MiR-1270 has been reported in thyroid cancer [24] and osteosarcoma [25] whereas it was not studied in LUAD.

Here, we analyzed the interaction between circ-SOX4 and miR-1270 by performing bioinformatics analysis and mechanism experiments. Consistently, the downstream mRNA and signaling pathway were explored. In summary, this study unveiled that circ-SOX4 promotes LUAD development via targeting miR-1270/PLAGL2 axis and activating WNT pathway, which might be helpful for exploring the new strategies to treat patients with LUAD.

## Materials and methods

### Clinical tissue specimens

Total LUAD tissues and adjacent normal tissues were obtained from China-Japan Union Hospital of Jilin University from May 2016 to August 2018. Before surgical resection, all involved participants signed informed consent. Afterwards, samples were frozen and stored at − 80 °C for further use. The procedures of this study were authorized by China-Japan Union Hospital of Jilin University.

### Microarray analyses

To begin with, RNase R (Epicentre, Inc., WI, USA) was applied to treat with total RNAs for removing linear RNAs and enriching circRNAs. Then, we amplified the enriched circRNAs and thereby transcribed these circRNAs into fluorescent cRNA using a random priming method (Arraystar, MD, USA). Afterwards, the labeled cRNAs were mixed into the Arraystar Human circRNA Array V2 (8 × 15K, Arraystar). After the slides being rinsed, the Agilent G2565CA Microarray Scanner System (Agilent Technologies, CA, USA) was utilized to scan the arrays. Agilent Feature Extraction software was employed to process the acquired array images. Quantile normalization was conducted by Arraystar software. Fold-change filtering identified differently expressed circRNAs between two groups.

### Cell culture and transfection

LUAD cell lines (A549,SPC-A1,H1299, PC-9; ATCC) and one normal human lung epithelial cell line (BEAS-2B; ATCC) were cultured in RPMI1640 medium (Gibco), supplemented with 10% fetal bovine serum (FBS; Gibco/Invitrogen Inc., USA) at 37 °C with 5% humidified CO_2_. For RNase R treatment, RNA was cultured at 37 °C for 15 min with or without RNase R (Epicentre Technologies, USA).

GenePharma (Shanghai, China) offered shRNAs targeting circ-SOX4 (sh-circ-SOX4#1/2) and negative control (sh-NC), pcDNA3.1 as well as pcDNA3.1/PLAGL2. MiR-1270 inhibitor, inhibitor control (NC inhibitor), miR-1270 mimics and mimics control (NC mimics) were also acquired from GenePharma. Lipofectamine 2000 Reagent (Invitrogen) was applied to conduct cell transfection in line with the protocols of the manufacturer.

### qRT-PCR

Under the suggestions of manufacturer, total RNA extracted from LUAD cells was isolated with TRIzol reagent (Invitrogen). The isolated RNAs (1 μg) were reverse-transcribed into cDNA by the use of TaqManTM Advanced miRNA cDNA Synthesis Kit (Waltham, MA, USA) or the reverse transcription kit (Takara, Otsu, Japan). qRT-PCR was carried out by employing the StepOneTM Real-Time PCR System and the SYBR^®^ Green Mixture (Takara). GAPDH or U6 was acted as the internal control. The 2^−ΔΔCt^ method was utilized to quantify relative expression levels.

### CCK-8 assay

The proliferation of LUAD cell was detected through a Cell Counting Kit-8 (Dojindo Molecular Technologies, Japan). 1 × 10^3^ cells were added into a 96-well plate, followed by the incubation of 24, 48, 72 and 96 h. Afterwards, each well was added with 10 μl CCK-8 solution, followed by another 4 h of incubation. The absorbance was detected at 450 nm using a spectrophotometer (Olympus, Japan).

### Colony formation assay

LUAD cells were placed in 6-well plates for an incubation of 2 weeks. Then, the methanol was utilized to fix the colonies for 30 min, and 0.1% crystal violet was applied to stain the colonies for 20 min as well as PBS was employed to wash the colonies three times. The number of colonies was manually counted.

### Wound healing assay

Wound-healing assay was carried out to measure cell migration. To begin with, cells were trypsinized and added into 6-well plates. After 12 h, pipette tip was employed to make an artificial wound. 0 h and 24 h after wounding, the wound width was examined, respectively.

### Transwell assay

A 24-well transwell chamber (8 mm, Corning Life Sciences, Corning, USA) was utilized in transwell assay. Matrigel (BD Biosciences, USA) coated the upper surface of the membrane. The top chamber was introduced with a total of 1 × 10^3^ cells suspended in serum-free medium, and 10% FBS was added into the lower chamber. Being cultured for 24 h, cells on the upper membrane surface were carefully removed. Afterwards, methanol was utilized to fix the lower membrane surface and 0.1% crystal violet was applied to stain the surface. Finally, a microscope was applied to calculate the number of invaded cells.

### Subcellular fractionation assay

PARIS™ Kit (Ambion, Austin, TX, USA) was applied to isolate nuclear fraction from cytoplasm fraction under the instructions of the manufacturer. A549 and SPC-A1 cell lines were eluted twice, dissolved in cell fraction buffer. Afterwards, the supernatant was collected after centrifugation. Subsequently, PBS was utilized to rinse the left lysate for five times with and then the lysate was centrifuged. qRT-PCR analyzed the extracted RNAs, normalizing to U6 (nucleus control) and GAPDH (cytoplasm control).

### RNA immunoprecipitation (RIP) assay

To begin with, A549 and SPC-A1 cells were dissolved in RIP lysis buffer. Subsequently, they were incubated with RIP buffer that contains magnetic beads coated with Ago_2_ antibodies (Abcam, Cambridge, MA, USA) or IgG antibodies at 4 °C overnight. RNA was then purified and quantified by qRT-PCR.

### Luciferase reporter assays

The circ-SOX4-WT, circ-SOX4-Mut-1 and circ-SOX4-Mut-2, PLAGL2-WT, PLAGL2-Mut reporters were acquired from GeneArt™ Site-Directed Mutagenesis System (Thermo Fisher Scientific). Lipofectamine 2000 was utilized to co-transfect miR-1270 mimics or NC mimics with the constructed luciferase reporters in A549 or SPC-A1 cells. Luciferase activities were evaluated with a Dual Luciferase Assay Kit (OMEGA Engineering Inc.) after 48 h of incubation.

Biovector NTCC Ltd (Beijing, China) offered the TOP and FOP flash luciferase reporter vectors. Sh-NC, sh-circ-SOX4 or different concentration of pcDNA3.1/circ-SOX4 or was co-transfected with TOP Flash and FOP flash reporters into A549 or SPC-A1 cells with the employment of Lipofectamine 2000. After 48 h, Dual Luciferase Assay Kit was applied to evaluate the activity of Wnt/β-catenin signaling pathway.

### RNA pull-down assay

Circ-SOX4 RNAs were transcribed in vitro and biotin-labeled with Biotin RNA Labeling Mix (Roche). Biotinylated RNAs were cultured with protein extracted from A549 or SPC-A1 cells. Magnetic beads were utilized to mix with each binding reaction, and then the beads were rinsed with washing buffer. SDS-PAGE was employed to separate the circRNA-interacting proteins. After the gels being silver stained, the proteins were subjected to Western blot analysis.

### Western blot

Proteins were obtained from cell lysis, and then separated by SDS-PAGE. Later, they were transferred onto PVDF membranes. 5% defatted milk was applied to seal the immunoblots for 1 h. Next, primary antibodies were used to incubate with the membranes at 4 °C overnight and then the membranes were cultured with secondary antibodies at room temperature for another 1 h. Finally, the immunoblots were detected with BioImaging Systems (BIO-RAD, CA, USA).

### Xenograft tumors in nude mice

Male nude BALB/c mice aged 4–5 weeks and weighted 20 g were provided from Vital River Laboratory Animal Technology (Beijing, China). SPC-A1 cells transfected with sh-circ-SOX4 or sh-NC were subcutaneously injected into nude mice. The volume of tumor xenografts that formed in nude mice was detected every 4 days. Four weeks later, all the animal experimental protocols were approved by China-Japan Union Hospital of Jilin University.

### Immunohistochemical analysis

The paraffin-embedded tissues were deparaffinized, rehydrated and treated by 0.3% H_2_O_2_ for antigen retrieval. For staining, the tissues were treated with Ki-67 antibodies (Abcam, Cambridge MA). Ruptured by nuclear membrane for 30 min with 0.1% Triton X-100 and blocked with 5% normal donkey serum, slides were separately incubated with primary antibody and secondary antibody. The results were visualized with Olympus BX 41 Microscope (Olympus Corporation, Japan).

### Statistical analysis

SPSS 21.0 software (IBM, Armonk, USA) was utilized for data analyses. Student’s t-test was applied for evaluating differences of two groups and one-way ANOVA for more than two groups. Data were confirmed as mean ± standard deviation (SD). P < 0.05 was deemed as statistically significant. Each experiment was repeated three times.

## Results

### Circ-SOX4 is highly expressed and knockdown of it hampers cell growth in LUAD

**Fig. 1:**
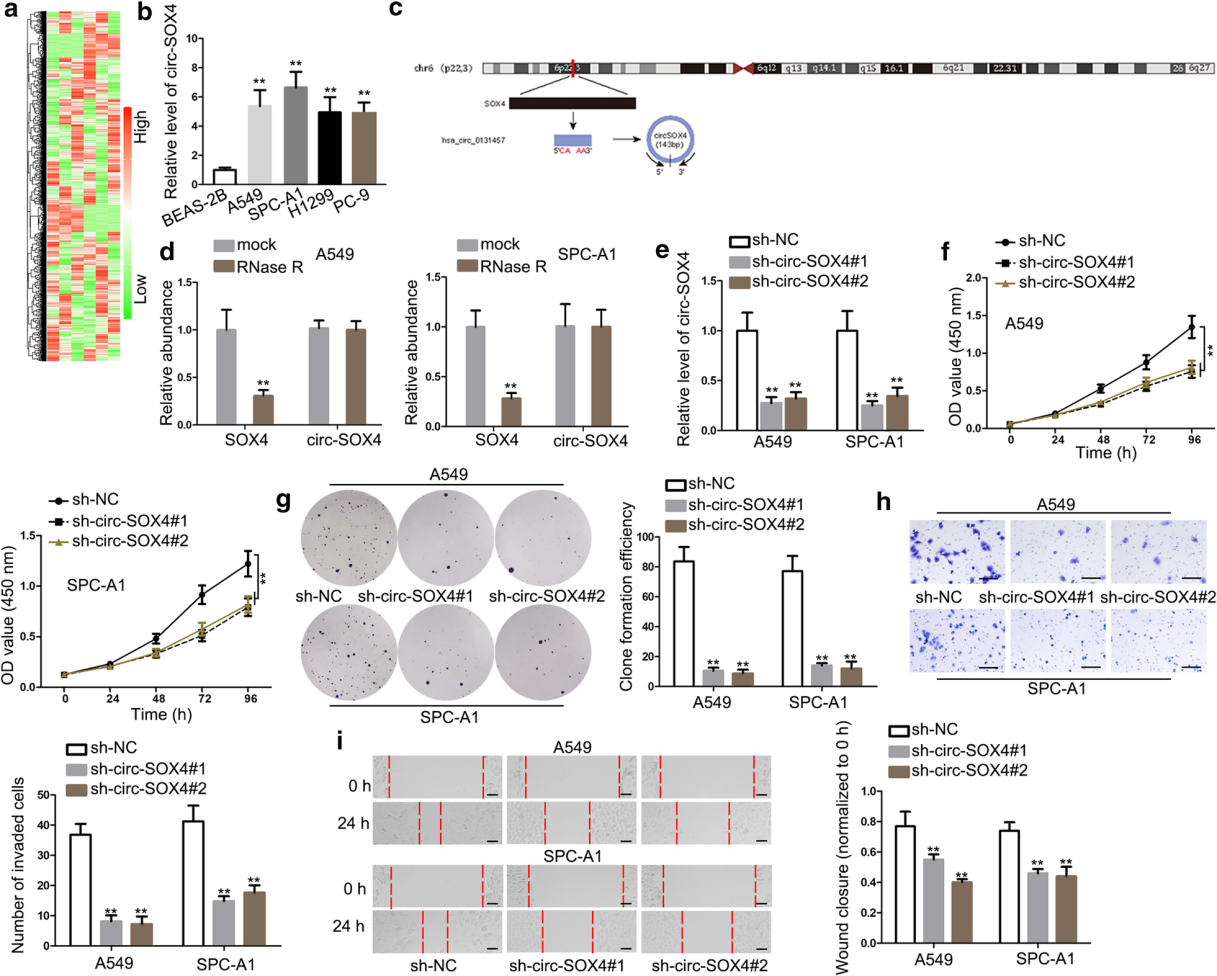
Circ-SOX4 is upregulated in LUAD and promotes cell growth in LUAD. **a** Heatmap displayed the dysregulated circRNAs in LUAD tissues and adjacent normal tissues. **b** qRT-PCR examined circ-SOX4 expression in LUAD cell lines (A549,SPC-A1 H1299 and PC-9) and normal human lung epithelial cell line (BEAS-2B). **c** Schematic diagram of the genomic location and splicing pattern of circ-SOX4. **d** The stability of circ-SOX4 and linear SOX4 in LUAD cells treated with RNase R (normalized to mock treatment) was assessed by qRT-PCR. **e** qRT-PCR examined the transfection efficiency of sh-circ-SOX4 in LUAD cells. **f**–**g** CCK8 and colony formation assays were carried to measure cell proliferation in sh-circ-SOX4 transfected cells. **h** Cell invasion was examined by transwell assay upon circ-SOX4 knockdown. Scale bar = 50 μm. **i** Cell migration in cells transfected sh-circ-SOX4 was evaluated by wound healing assay. Scale bar = 100 μm. **P < 0.01

**Fig. 2:**
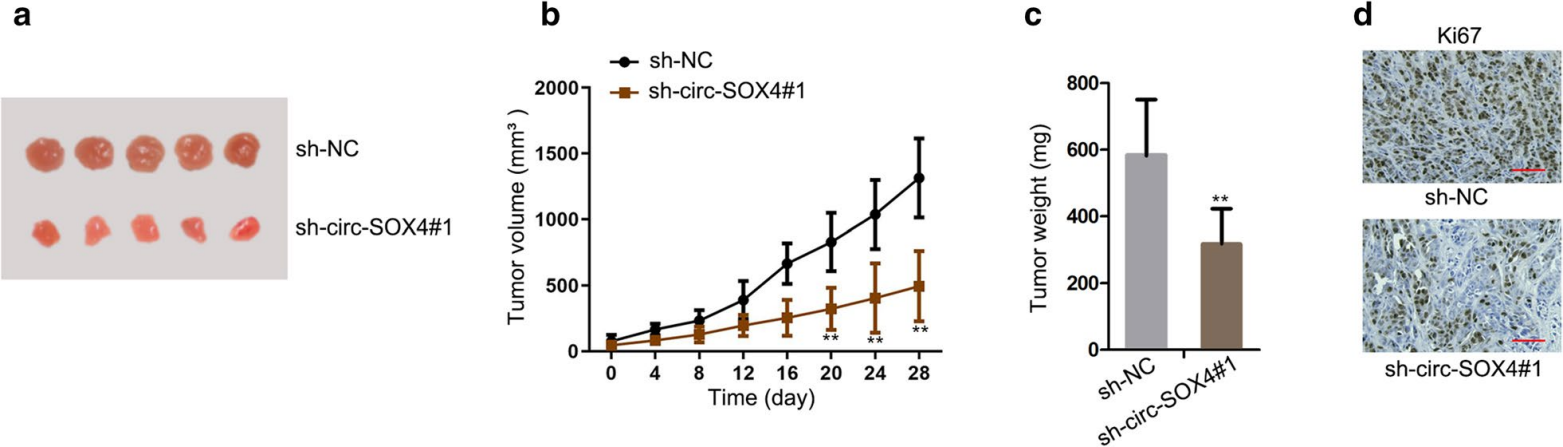
Circ-SOX4 promotes LUAD cell growth in vivo. **a** Tumors removed from mice in two groups were presented. **b**, **c** The tumor volume and weight in the groups of sh-NC and sh-circ-SOX4 were showed. **d** The expression of ki-67 was detected by IHC assay. **P < 0.01

To investigate the potential functional circRNAs in LUAD, microarray analysis was utilized to assess the expression of circRNAs in LUAD tissues and adjacent non-tumor tissues (Fig. 1a). Then circ-SOX4, which was mostly significantly upregulated in LUAD tissues, was chosen to conduct qRT-PCR assay. And one normal lung epithelial cell line (BEAS-2B) acted as a control. The result displayed that circ-SOX4 expression was dramatically upregulated in LUAD cell lines (A549, SPC-A1, H1299 and PC-9) (Fig. 1b). Additionally, schematic diagram displayed the genomic location and splicing pattern of circ-SOX4 (Fig. 1c). Moreover, the fragment of linear SOX4 mRNA was digested by RNase R, whereas circ-SOX4 remained after the treatment of RNase R (Fig. 1d). Subsequently, several experiments were applied to probe the biological function of circ-SOX4 in LUAD. With the employment of qRT-PCR analysis, a notably lower expression of circ-SOX4 was observed in LUAD cells with the transfection of sh-circ-SOX4 than that in sh-NC-transfected cells (Fig. 1e). Importantly, we found that the mRNA expression of SOX4 showed no evident changes in cells transfected with sh-circ-SOX4 (Additional file 1: Figure S1A). Later, CCK-8 and colony formation assays showed that circ-SOX4 deficiency repressed the proliferation ability of A549 and SPC-A1 cells (Fig. 1f–g). Afterwards, knockdown of circ-SOX4 weakened the invasion capability of A549 and SPC-A1 cells (Fig. 1h). Besides, the migration of A549 and SPC-A1 cells was repressed by circ-SOX4 knockdown (Fig. 1i). Further, we performed in vivo experiments to assess the effect of circ-SOX4 on the tumor growth of LUAD. As demonstrated in Fig. 2a, tumors derived from circ-SOX4 silenced LUAD cell were remarkably smaller than those in NC group. Consistently, the tumor volume and weight were also smaller in sh-circ-SOX4 group compared with sh-NC group (Fig. 2b, c). Results of IHC assay revealed that ki-67 expression was downregulated after circ-SOX4 was stably silenced (Fig. 2d). To sum up, circ-SOX4 is highly expressed and circ-SOX4 depletion represses cell proliferation, invasion, migration and tumor growth in LUAD.

### Circ-SOX4 sponges miR-1270 in LUAD

**Fig. 3:**
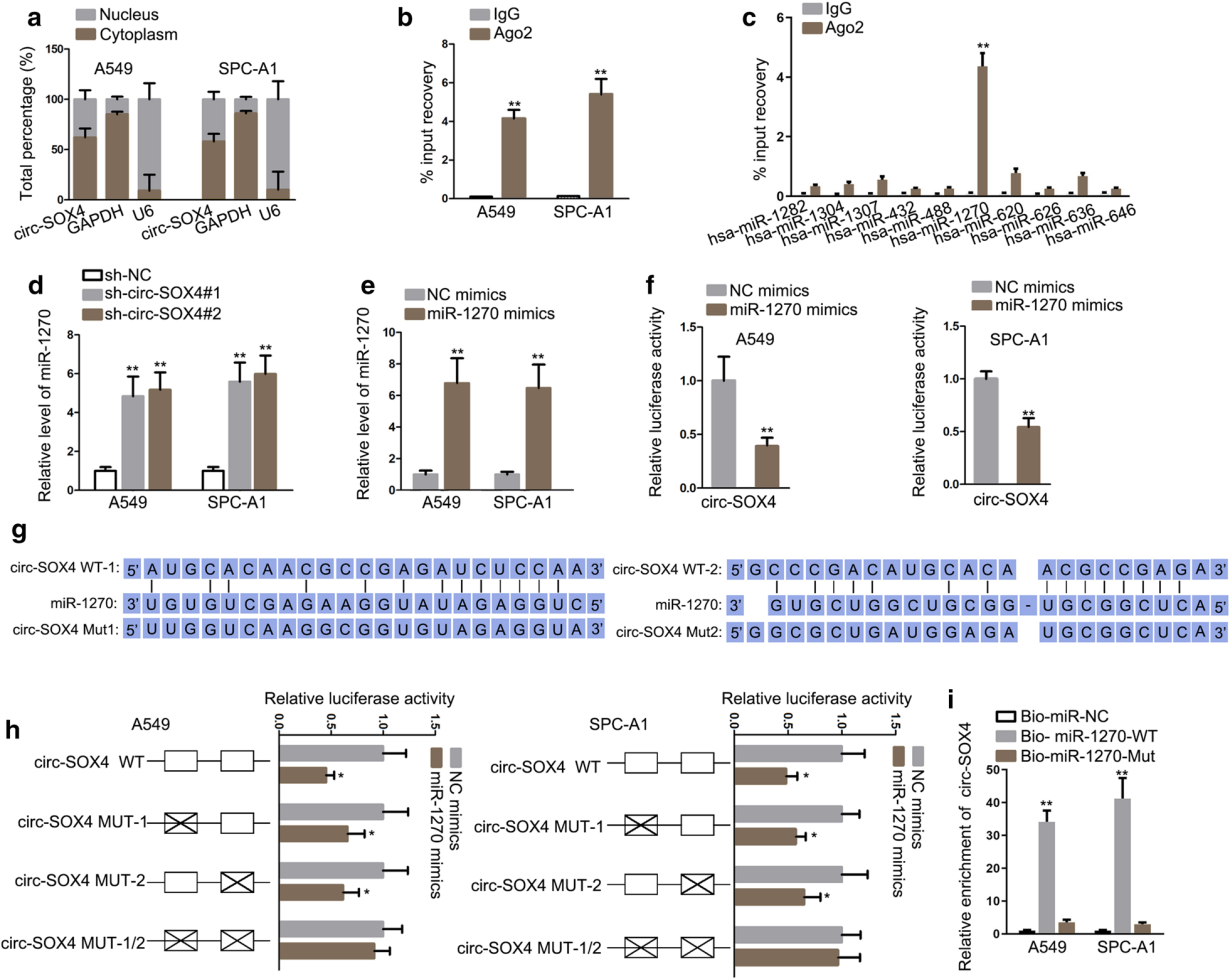
Circ-SOX4 sponges miR-1270. **a** Subcellular fraction assay was carried out to examine the distribution of circ-SOX4 in nuclear and cytoplasm of A549 and SPC-A1 cells. **b** RIP assay was performed to confirm that circ-SOX4 could bind with RNA-induced silencing complex (RISC). **c** Several miRNAs that possess the potential of binding with circ-SOX4 were predicted by circular RNA interactome database and screened out by RIP assay **d** The influence of circ-SOX4 deficiency on miR-1270 expression was evaluated by qRT-PCR. **e** The overexpression efficiency of miR-1270 was examined in LUAD cells. **f** The effect of miR-1270 overexpression on the luciferase activity of reporter containing circ-SOX4 sequence was assessed by luciferase reporter assay. **g** The predicted binding sites between miR-1270 and circ-SOX4 were depicted. **h**–**i** Luciferase reporter and RNA pull down assays were conducted to testify the binding ability between miR-1270 and circ-SOX4. *P < 0.05, **P < 0.01, ***P < 0.001

With the intention of further probing the molecular mechanism of circ-SOX4 in LUAD, subcellular fractionation assay was conducted to determine the distribution of circ-SOX4 in cytoplasm and nucleus, and the result suggested that circ-SOX4 mainly localized in cytoplasm (Fig. 3a). In addition, circ-SOX4 was found to enrich in Ago_2_ antibody group but not in IgG antibody group (Fig. 3b). Existing investigations have confirmed that circRNAs contribute to cancer progression by sponging specific miRNA [26, 27]. Therefore, circular RNA interactome database was searched to predict potential miRNAs for circ-SOX4, and miR-1282, miR-1304, miR-1307, miR-432, miR-488, miR-1270, miR-620, miR-626, miR-636 and miR-646 were found. RIP analysis delineated that miR-1270 was the most enriched in Ago_2_ antibody group compared with other miRNAs (Fig. 3c). Herein, miR-1270 was chosen to do the following experiments. qRT-PCR assay indicated that circ-SOX4 depletion significantly increased miR-1270 expression in LUAD cells (Fig. 3d). In addition, we tested the transfection efficiency of miR-1270 mimics in LUADA cells. As a result, miR-1270 mimics remarkably increased miR-1270 expression (Fig. 3e). Furthermore, the luciferase activity of vector containing circ-SOX4 sequence observably decreased by miR-1270 mimics, suggesting the potential interaction between circ-SOX4 and miR-1270 (Fig. 2f). Through searching starBase, it was predicted that miR-1270 had two binding sites for circ-SOX4 (Fig. 3g). Luciferase reporter assay further confirmed that the circ-SOX4 interacted with miR-1270 in the above two binding sites (Fig. 3h). What’s more, RNA pull down assay validated that circ-SOX4 bind to miR-1270 (Fig. 3i). Overall, circ-SOX4 functions as a sponge for miR-1270 in LUAD.

### PLAGL2 is a target gene of miR-1270 in LUAD

**Fig. 4:**
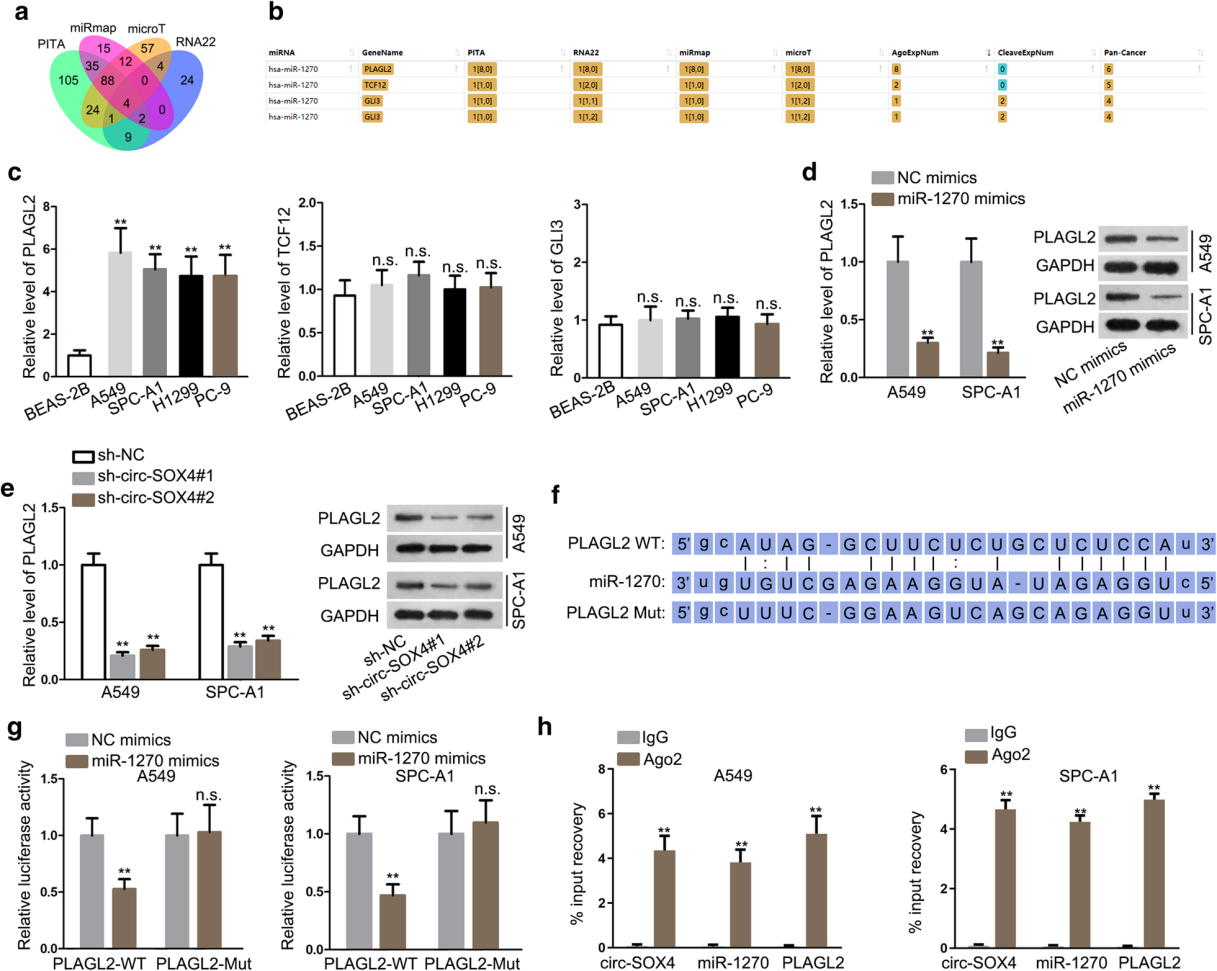
MiR-1270 could bind with its downstream mRNA PLAGL2. **a** Venn diagram displays the overlaps of the analysis performed in this study. **b** The potential mRNAs that could bind with miR-1270 were searched from PITA, RNA22, miRmap and microT databases. **c** qRT-PCR examined the expressions of predicted mRNAs (PLAGL2, TCF12 and GLI3) in LUAD cell lines and normal human lung epithelial cell line. **d**–**e** The mRNA and protein levels of PLAGL2 in miR-1270 mimics-transfected or sh-circ-SOX4-transfected cells were evaluated by qRT-PCR and western blot assay, respectively. **f** The predicted binding site between miR-1270 and PLAGL2 were shown. **g** Luciferase reporter assay was conducted to prove the binding ability between miR-1270 and PLAGL2. **h** RIP assay was performed to testify that circ-SOX4, miR-1270 and PLAGL2 in RISC. **P < 0.01

Afterwards, through searching PITA, RNA22, miRmap and microT databases, the potential target gene of miR-1270 was displayed by Venn diagram and the predicted mRNAs were PLAGL2, TCF12 and GLI3 (Fig. 4a, b). qRT-PCR analysis showed that PLAGL2 expression was evidently upregulated in LUAD cell lines whereas the expression of TCF12 and GLI3 showed no remarkable difference (Fig. 4c). qRT-PCR analysis and western blot assay depicted that PLAGL2 mRNA and protein expressions were significantly reduced by miR-1270 overexpression or circ-SOX4 silencing (Fig. 4d, e). As displayed in Fig. 4f, miR-1270 had a binding site for PLAGL2. Then, luciferase reporter assay demonstrated that the luciferase activity of PLAGL2-WT was observably decreased by miR-1270 mimics while that of PLAGL2-Mut depicted no notable change (Fig. 4g). Finally, circ-SOX4, miR-1270 and PLAGL2 were all aggregated in Ago_2_-conjugated beads via RNA pull down assay (Fig. 4h). Taken together, PLAGL2 is a target gene of miR-1270 in LUAD.

### Circ-SOX4 promotes LUAD cell growth by sponging miR-1270 and upregulating PLAGL2 expression

**Fig. 5:**
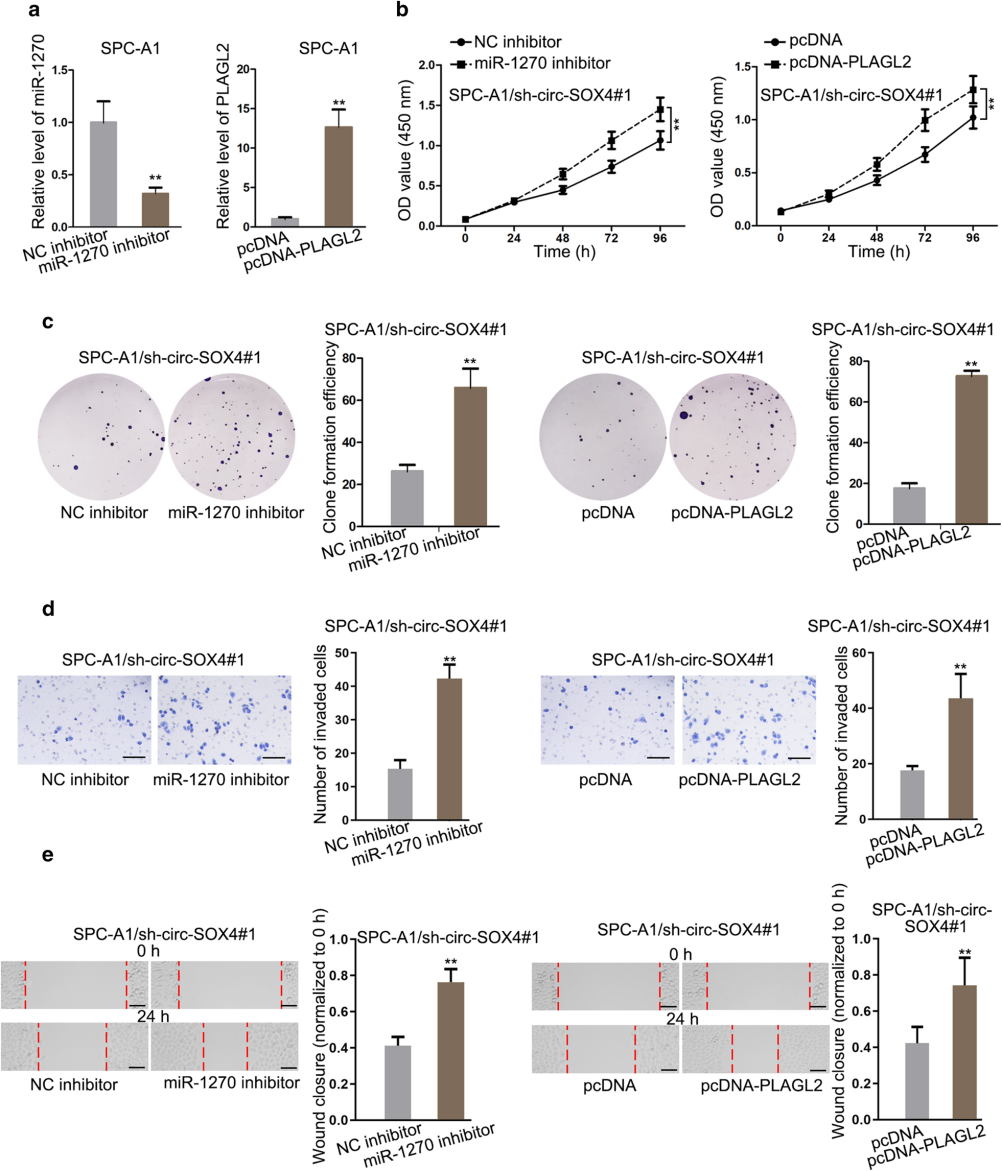
Circ-SOX4 promotes LUAD progression through sponging miR-1270 and upregulating PLAGL2. **a** qRT-PCR measured the transfection efficiency of miR-1270 inhibitor and pcDNA-PLAGL2. **b**, **c** Cell proliferation was examined by CCK8 and colony formation assays. **d** Transwell assay was carried out to measure cell invasion in LUAD. Scale bar = 50 μm. **e** Wound healing assay evaluated cell migration in transfected cells. Scale bar = 100 μm. **P < 0.01

To testify whether circ-SOX4 promotes LUAD development by sponging miR-1270 and upregulating PLAGL2 expression, rescue assays were carried out. Before conducting rescue experiments, the transfection efficiency of miR-1270 inhibitor and pcDNA-PLAGL2 were measured by qRT-PCR in APC-A1 cells. The results depicted that miR-1270 inhibitor evidently inhibited miR-1270 expression and PLAGL2 expression was conspicuously enhanced by transfecting pcDNA-PLAGL2 (Fig. 5a). Then, CCK-8 and colony formation assays demonstrated that miR-1270 suppression or PLAGL2 overexpression countervailed circ-SOX4 depletion-mediated inhibitive effect on cell proliferation (Fig. 5b, c). Besides, inhibitory function caused by circ-SOX4 deficiency on cell invasion was recovered by miR-1270 suppression or overexpressed PLAGL2 (Fig. 5d). What’s more, it was confirmed by wound healing assay that the inhibition of miR-1270 or PLAGL2 upregulation offset the inhibitive effect of circ-SOX4 knockdown on cell migratory ability (Fig. 5e). In summary, circ-SOX4 promotes LUAD cell proliferation, invasion and migration by sponging miR-1270 and upregulating PLAGL2 expression.

### Circ-SOX4/miR-1270/PLAGL2 signal axis activates WNT pathway in LUAD

**Fig. 6:**
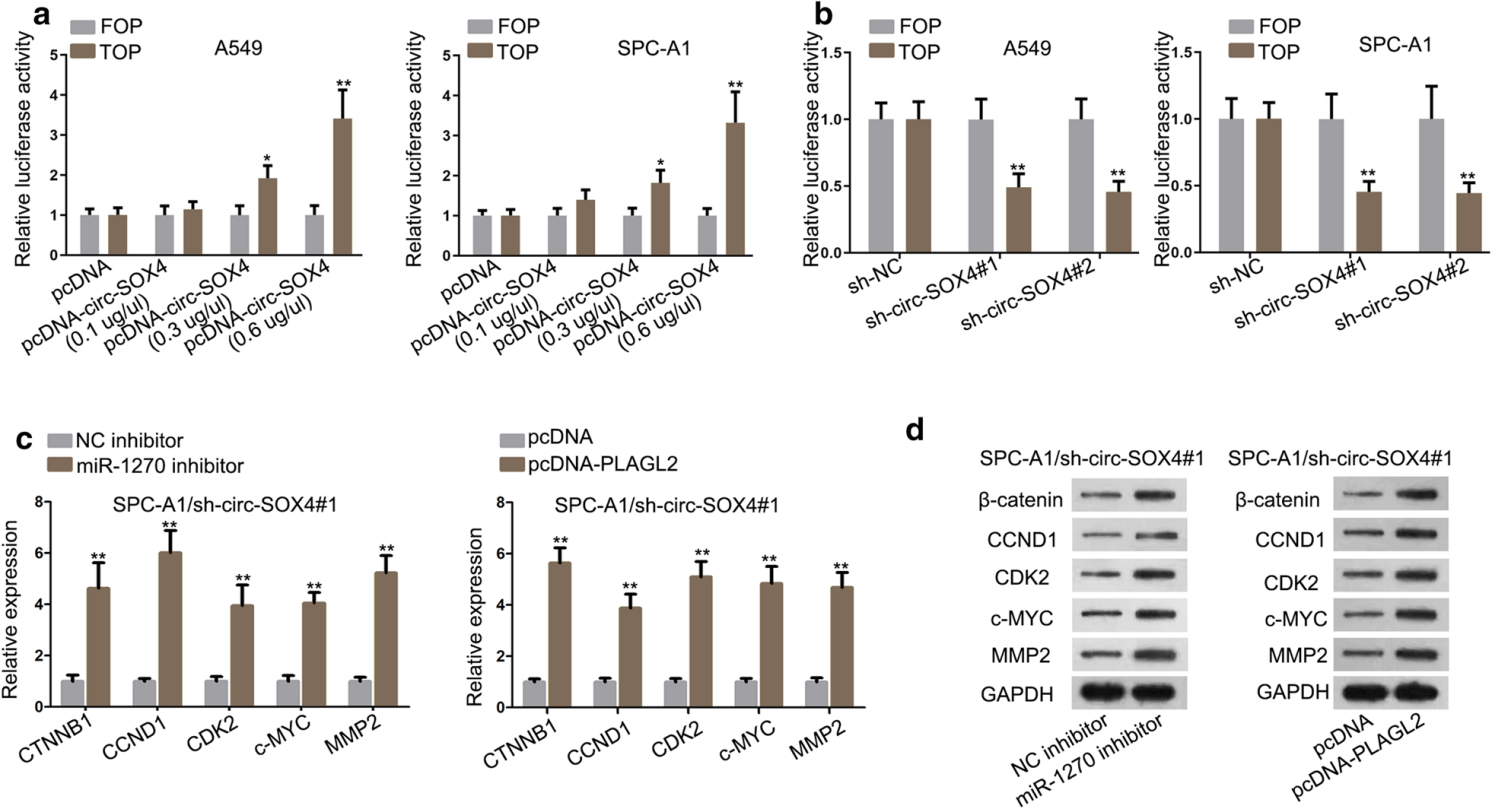
Circ-SOX4 promotes LUAD progression through miR-1270/PLAGL2/WNT/β-catenin signaling pathway. **a** TOP/FOP flash assay evaluated the influences of different concentration of pcDNA3.1/PLAGL2 on the luciferase activity of TOP and FOP reporters. **b** The luciferase activities of TOP and FOP reporters in sh-NC and sh-circ-SOX4 groups were assessed by TOP/FOP flash assay. **c**, **d** qRT-PCR and western blot assay, respectively evaluated the level of WNT pathway-related mRNAs and proteins in SPC-A1/sh-circ-SOX4 cells. *P < 0.05, **P < 0.01

According to existing literature, PLAGL2 has been reported to activate WNT pathway in colorectal adenocarcinoma [28]. To find out whether circ-SOX4/miR-1270/PLAGL2 signal axis activated WNT pathway in LUAD, the following experiments were performed. Through TOP/FOP flash assay, we observed that the activity of WNT pathway was effectively promoted with the increasing concentration of pcDNA-circ-SOX4 (Fig. 6a). Further, the activity of WNT pathway was restrained by circ-SOX4 deficiency (Fig. 6b). Lastly, qRT-PCR and western blot assays displayed that repressed miR-1270 or upregulated PLAGL2 reserved circ-SOX4 depletion-mediated effect on the mRNA and protein expressions of CTNNB1 (β-catenin), CCND1, CDK2, c-MYC and MMP2, which were known as the relative genes of WNT pathway (Fig. 6c, d). Further, we detect the protein level of CD44, E-cadherin, and N-cadherin for checking epithelial-mesenchymal transition (EMT). The results displayed that the inhibition of miR-1270 or overexpressed PLAGL2 increased the protein level of CD44 and N-cadherin whereas reduced that of E-cadherin in sh-circ-SOX4 transfected cells (Additional file 1: Figure S1B). According to previous reports, we found that CD44 and EMT were closely correlated with WNT pathway [29–32]. All the findings suggest that circ-SOX4/miR-1270/PLAGL2 signal axis activates WNT pathway in LUAD.

## Discussion

Despite various advances in the treatment of LUAD, it is still one of the main reasons of death globally [33]. For the past few years, the cases of LUAD increased annually and ascended to the highest morbidity kind of NSCLC [34]. A growing number of studies have indicated that the aberrant expression of circRNAs displayed regulatory roles in diverse cancers [35–38]. In the current investigation, we chose an upregulated circRNAs in LUAD tissues through a microarray analysis. To our knowledge, circ-SOX4 was firstly investigated in LUAD. Moreover, we observed high expression of circ-SOX4 in LUAD tissues and cell lines. Functionally, high level of circRNAs might exert regulatory functions in LUAD. As recently reported, dysregulation of circRNAs can contribute to the malignant behaviors of human cancer cells. For instance, high level of hsa_circ_0000515 acts as a tumor promoter in cervical cancer through miR-326/ELK1 axis [39]. CircRNA-ENO1 and its linear mRNA ENO1 promotes glycolysis and tumor formation in LUAD [40]. Circular form of ERBB2 promotes the malignant process in gallbladder cancer [41]. In this study, we also designed and performed functional experiments to determine the role of circ-SOX4 in LUAD cellular processes. After silencing of circ-SOX4, we determined that circ-SOX4 knockdown hampered cell proliferation, invasion and migration in LUAD. In vivo experiments showed that silenced circ-SOX4 was correlated with the decreased tumor size, volume, weight, and ki-67 expression. In consequence, it was confirmed that circ-SOX4 accelerated the development of LUAD.

Mechanistically, circRNAs have been reported as sponges for miRNAs, thus functioning as promoter or inhibitor in carcinogenesis. In our current study, we observed that circ-SOX4 was predominantly located in the cytoplasm of LUAD cells. Based on bioinformatics analysis, miRNAs that were potentially interacted with circ-SOX4 were predicted. Among which, miR-1270 had highest enrichment in LUAD cells. Thus, it was selected as the next research object. MiR-1270 was reported in a previous study as its tumor-suppressive role [42]. In this research, it was found that circ-SOX4 could directly bind to miR-1270 and miR-1270 expression was negatively regulated by circ-SOX4 in LUAD. Briefly, circ-SOX4 facilitated LUAD development by serving as a sponge for miR-1270.

Previous studies have certified that circRNAs affect the development of cancer by sponging miRNAs to regulate mRNAs [43, 44]. For illustration, circ-GFRA1 sponges miR-34a and promotes the progression of breast cancer by modulating GFRA1 [45]. Hsa-circ-0045714 regulates chondrocyte viability and apoptosis by enhancing miR-193b expression and targeting IGF1R [46]. According to mechanism investigation, we confirmed that miR-1270 could interact with PLAGL2. PLAGL2 has been confirmed as an oncogene in colorectal cancer [47] and bladder urothelial carcinoma [48]. According to the results in this study, PLAGL2 expression was negatively regulated by miR-1270 and positively regulated by circ-SOX4 in LUAD cells. Importantly, PLAGL2 was reported to activate WNT pathway [49]. Thus, we further investigated that circ-SOX4 could activate Wnt pathway via upregulation of PLAGL2. Through rescue assays, we found that miR-1270 repression or PLAGL2 elevation reversed the inhibitory role of circ-SOX4 silencing in LUAD cell growth and Wnt pathway.

## Conclusion

In conclusion, our study revealed that circ-SOX4 boosted LUAD progression by targeting miR-1270/PLAGL2 axis and activating Wnt pathway. Above results in present study might provide the meaningful revelation for investigating the novel therapeutic methods for LUAD patients.

## Supplementary information

**Additional file 1: Figure S1.** (A) SOX4 expression was tested in sh-circ-SOX4 transfected cells. (B) Western blot assay was performed to assess the protein levels of CD44, E-cadherin and N-cadherin. n.s.: no significance. **P < 0.01.

## Data Availability

Research data and material are not shared.

## Abbreviations

LUAD: lung adenocarcinoma
circRNA: circular RNA
NSCLC: non-small cell lung cancer
SOX4: SRY-box transcription factor 4
PLAGL2: PLAG1 like zinc finger 2
shRNA: short hairpin RNA
FBS: fetal bovine serum
NC: negative control
GAPDH: glyceraldehyde-3-phosphate dehydrogenase
CCK-8: cell counting kit-8
PBS: phosphate buffer saline
qRT-PCR: quantitative real-time polymerase chain reaction
IgG: immunoglobulin G
PVDF: polyvinylidene fluoride
SD: standard deviation
RISC: RNA-induced silencing complex
miRNAs: microRNAs
