# Supplementary figures and images for: Circ-SOX4 drives the tumorigenesis and development of lung adenocarcinoma via sponging miR-1270 and modulating PLAGL2 to activate WNT signaling pathway
Source: Cancer Cell Int. 2020 Jan 3;20:2. doi: 10.1186/s12935-019-1065-x (PMC6942331; doi:10.1186/s12935-019-1065-x)

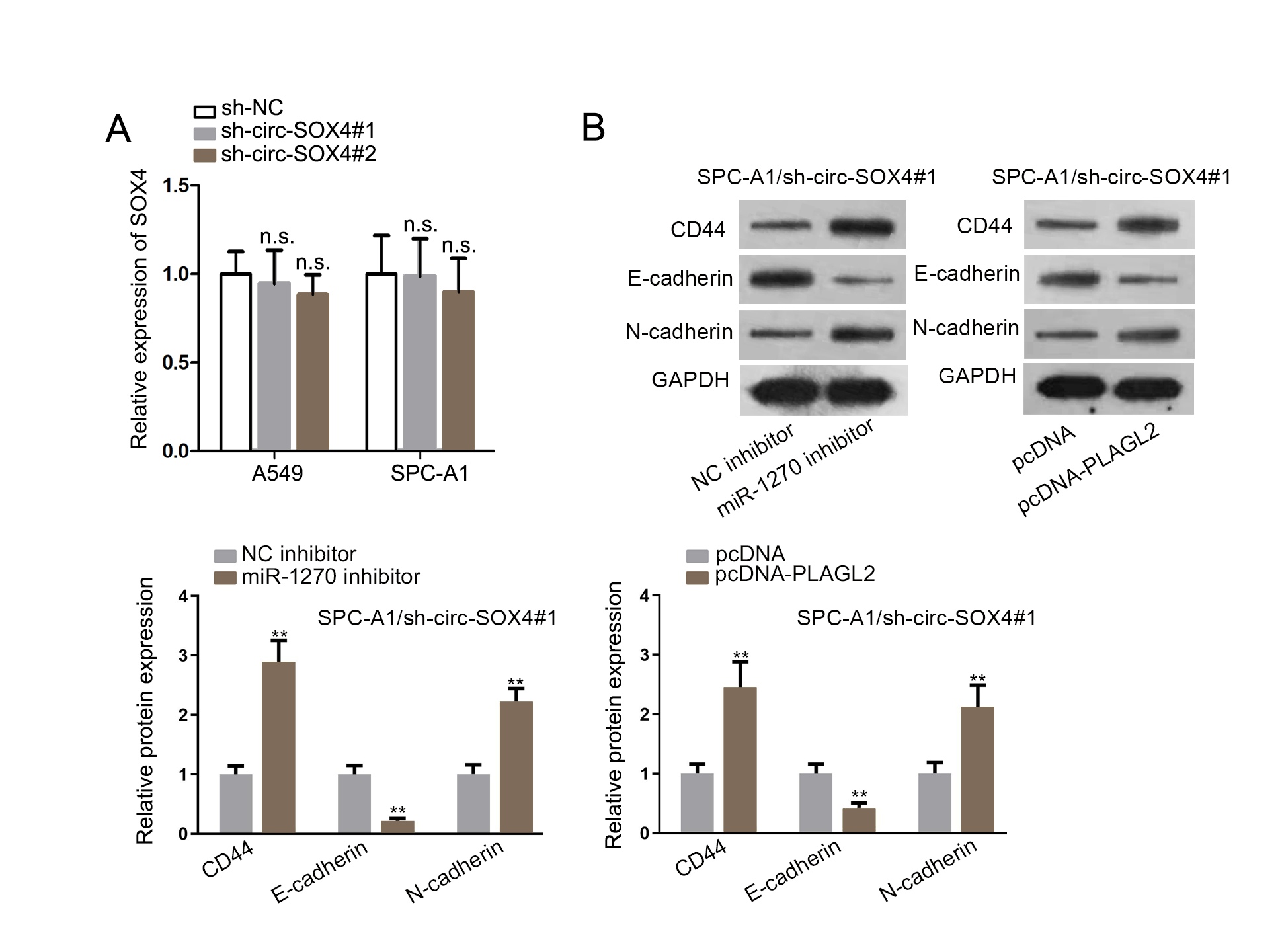

Supplement: Supplementary file 1 — Additional file 1: Figure S1. (A) SOX4 expression was tested in sh-circ-SOX4 transfected cells. (B) Western blot assay was performed to assess the protein levels of CD44, E-cadherin and N-cadherin. n.s.: no significance. **P < 0.01. [file 12935_2019_1065_MOESM1_ESM.tif]
